# A pilot study on lengthening potentials and biomechanical effects of double and triple hemisection on tendon with slide lengthening

**DOI:** 10.1038/s41598-023-30791-w

**Published:** 2023-03-09

**Authors:** T. Wang, H. Yu, Guo-fu Tian, Rui-xiang Zhao

**Affiliations:** 1 Burn and Plastic Surgery Department, Shenyang 242 Hospital, No.3 Leshan Road, Shenyang, 110000 China; 2 Graduate School, Shenyang Medical College, No.5 South Qi West Road, North Huanghe Street, Shenyang, 110000 China; 3 Department of Orthopedics, The First Hospital of China Medical University, 155 Nanjing North Street, Shenyang, 110000 China; 4 School of Mechanical Engineering, Shenyang University Of Technology, Shenyang, China

**Keywords:** Preclinical research, Biomedical engineering, Biological physics

## Abstract

The current study explored the slide-lengthening potentials of double and triple hemisections and the biomechanical effects of different inter-hemisection distances. Forty-eight porcine flexor digitorum profundus tendons were divided into double- and triple-hemisection groups (Groups A and B) and a control group (Group C). Group A was divided into Group A1 (distance between hemisections were the same as Group B) and Group A2 (distance between hemisections corresponded to the greatest distance between hemisections in Group B). Biomechanical evaluation, motion analysis, and finite element analysis (FEA) were performed. Failure load of intact tendon was significantly highest among groups. When the distance was 4 cm, the failure load of Group A increased significantly. When the distance between the hemisections was 0.5 or 1 cm, the failure load of Group B was significantly lower than Group A. Tendon elongation and failure load of Group B were significantly lower than those in Group A when the greatest distance between hemisections was the same. Consequently, Double hemisections had a similar lengthening ability to that of triple hemisections with the same distance, but better when the distances between extreme hemisections matched. However, the driving force for the initiation of lengthening may be greater.

## Introduction

Spastic paralysis presents with abnormal limb morphology and dysfunction, such as muscle spasm and joint contracture^1,2^. Tendon lengthening is a type of treatment for spastic paralysis, a movement disorder caused by brain and spinal cord injury^3^. Tendon lengthening is commonly used when physical therapy such as acupuncture, massage, or orthosis; oral or injection drugs^4,5^; and cervical nerve root transposition^6,7^, which is a newly proposed treatment; are not effective. Tendon lengthening including fractional lengthening, slide lengthening, and Z-shaped lengthening^8–17^ can reduce spastic muscle tension by changing tendon length^18^ and providing a sufficient range of motion. Clinically, fractional lengthening could be an effective method for moderate deformity, but not for severe deformity, as such cases require a muscle–tendon overlapping area^11,19,20^.

Z-shaped lengthening is commonly used for upper-limb tendon contracture^8–10,17^. The extension potential of the Z-shaped lengthening is considered “unlimited”^21^. However, the larger incisions and greater tendon damage associated with it can lead to more complications^22^. Slide lengthening can reduce incision size, shorten operation time, and decrease the incidence of incision complications^13^. Moreover, the collagen fibers of the treated tendon retain a certain continuity, which can help achieve better biomechanical effects^19,23^. However, slide lengthening is mainly used to lengthen the Achilles tendon with triple hemisections^15^. Its application in upper limb tendons has rarely been reported^19,20^. It has been suggested that slide lengthening with double hemisections as a treatment for severe deformity of the upper extremities could lengthen tendons precisely to the required degree and avoid inequality in finger position^20^. Clinically, the average number of incisions required for tendon lengthening is 2 or 3^8–17^. However, the optimal number of hemisection planes and the optimal distance between them still needs to be determined. Consequently, the purpose of this study was to compare the slide lengthening potentials between the double and triple hemisection methods and to explore the biomechanical effects of different distances between the hemisection planes in those methods.

## Materials and methods

### Animals and preparation

**Figure 1:**
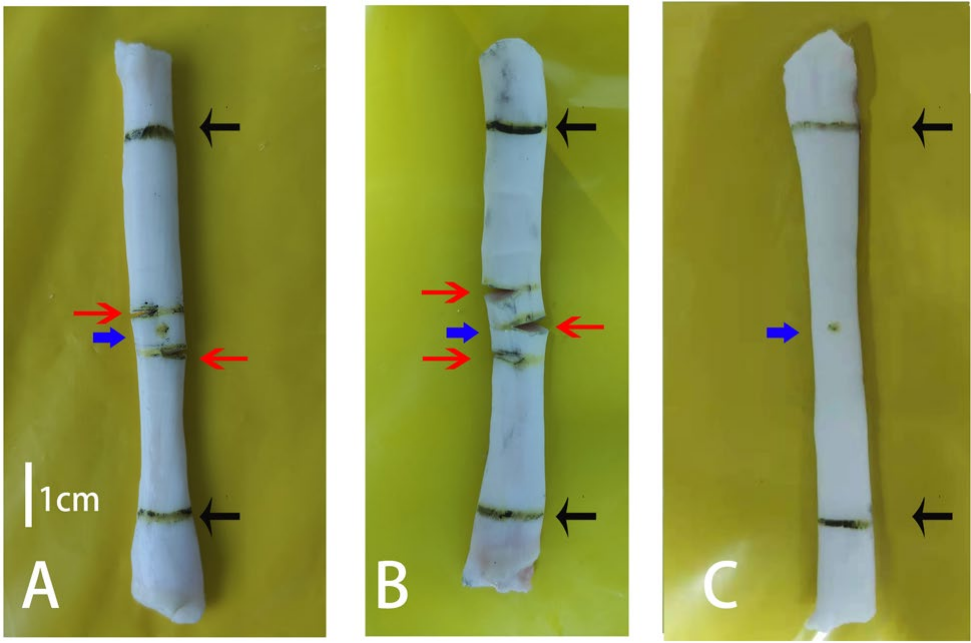
The model of tendon hemisection. (**A**) double hemisection tendon, (**B**) triple hemisection tendon, and (**C**) complete tendon. The distance between hemisections in A and B was 0.5 cm. The length of the test area of the tendons ( distance between the two clamp lines) was 6 cm. The central point, clamping line, and hemisection planes of the tendon are marked (indicated by blue, black, and red arrows, respectively). On the tendons with hemisections, incisions were cut to half or more of the cross-sectional area of the tendon. The central point and clamp lines are marked on the intact tendon.

**Figure 2:**
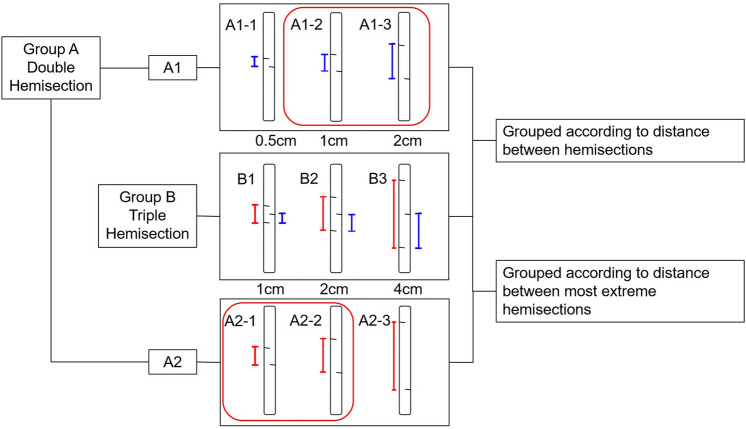
Schematic diagram of Group A and Group B. The red line represents the distance between the two farthest hemisections, whereas the blue line represents the distance between the hemisections. Group A was the double-hemisection group and Group B was the triple-hemisection group. Group A1 was divided into A1-1, A1-2 and A1-3 groups, corresponding to the distance between hemisections of 0.5, 1, and 2 cm, respectively. Group B was divided into B1, B2, and B3, corresponding to distances of 0.5, 1 and 2 cm between hemisections, respectively. Because of the different distances between the farthest hemisections and the possibility that the biomechanical properties of the tendons would be compromised, Group A2 was designed. The two incisions in Group A2 were in the plane of the most distal incisions of Group B, corresponding to the distance between the hemisections of 1 cm, 2 cm and 4 cm; they were divided into A2-1, A2-2 and A2-3 respectively. The two groups of tendons marked in the red box represent the same tendon.

Flexor digitorum profundus tendons were obtained from 48 porcine hind legs procured from a local butcher. After the muscle fibers and surrounding soft tissues were removed, the tendons were wrapped with gauze soaked in saline and stored at 0–4 °C. Subsequently, tendons were sectioned. Since the flexor digitorum profundus tendon is approximately 10 cm long^24^, areas 3 cm above and below the midpoint of the tendons were selected, meaning a 6 cm area was the test range. Subsequently, 2 cm of each end of the tendon was clamped. The tendon center, hemisection plane, and clamping line were marked according to the requirements of each group (Fig. 1). Group A underwent double hemisection, Group B underwent triple hemisection, and Group C was the control group (Fig. 2).

Limited by the length of the tendon test area, half of the tendon fibers were severed in the contralateral–ipsilateral–contralateral pattern according to the distance between hemisections, which were performed at 0.5, 1 and 2 cm points in Group B. Half of the tendon fibers were severed in the contralateral–ipsilateral pattern in Group A, which was divided into Groups A1 and A2. Group A1 had the same distance between hemisections as Group B, meaning that the distances between hemisections in Group A1 were 0.5, 1, and 2 cm. Group A2 corresponded to the largest distance between hemisections in Group B; that is, the distances between the hemisections in Group A2 were 1, 2 and 4 cm. (Figs. 1, 2). After the models were constructed, the tendons were wrapped in saline-soaked gauze and placed in sealable plastic bags^25,26^. They were then stored at − 20 °C to confirm that the biomechanical properties of the tendons did not change significantly within 3 months^27^.

### Biomechanical evaluation

**Figure 3:**
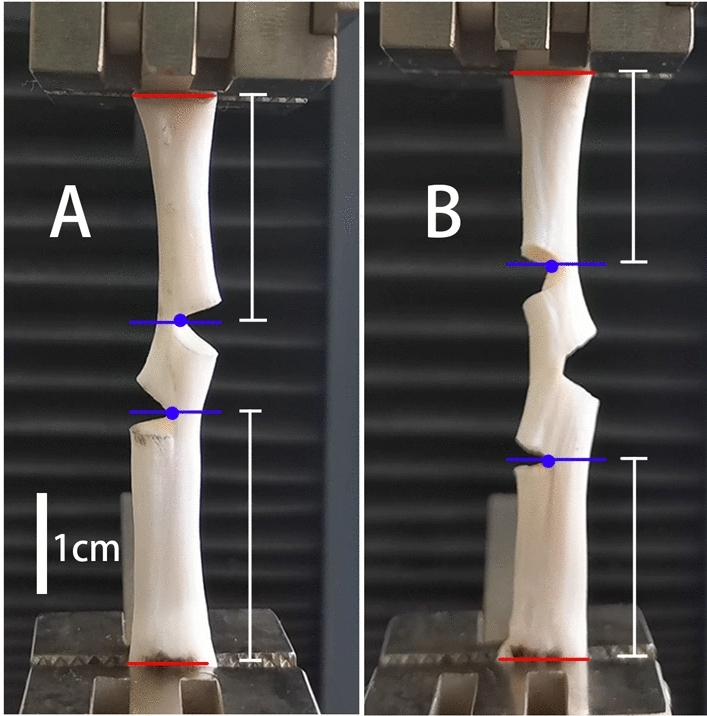
Tendons in the biomechanical tensile test. (**A**) double hemisection. (**B**) triple hemisection. The distance between the incisions in A and B was 1 cm. Red line: clamping line. Blue lines: planes of the hemisections farthest apart. Blue dot: vertex of angle formed by hemisection deformation. The plane of the hemisection was marked as a line passing through the vertex of the angle formed by hemisection deformation and perpendicular to the long axis of the tendon. The distance between the red and blue lines (white line) is the distance between the clamp line and nearest hemisection.

Both ends of the tendons were fixed on a microcomputer-controlled electromechanical universal testing machine (Wance Testing Machine Co., Shenzhen, China, ETM203A) along the clamping lines. The tendons were stretched at a constant speed of 20 mm/min^28^ until they were divided into two parts (Fig. 3). Failure occurred when the tendons in Groups A and B began to slide or when the tendons in Group C ruptured. The failure load, which was the force applied to the tendon when it was ruptured, and the elongation, which was the increased length of the tendon during stretching, were recorded. Simultaneously, the force–elongation curve (F-L curve) was measured. During the experiment, small amounts of saline were sprayed to keep the tendons moist^28^. Additionally, the temperature was monitored using a thermometer and maintained at 19 °C ensure that the mechanical properties of tendons were not affected.

### Motion analysis

The entire biomechanical test process was video-recorded. The video recording device (Mate 20, Huawei, Shenzhen, China) was placed square to the central point of the tendon, approximately 30 cm away, to record the two-dimensional movement of the tendon^28,29^. When the stretch test was started, the video recording was synchronized. The total length of the tendons in Groups A and B, when the tendons began to split and divide into two parts, and the distance between the clamp line and the nearest hemisection was recorded. Additionally, the total length of the tendons in Groups A and B and, when the failure load was reached, where the tendon was nearly divided into two parts or ruptured, was also tracked by motion analysis software (a frame-by-frame player; Kinovea, V0.8.27 [Free Software Foundation, Inc., Boston, MA, USA]).

### Finite element analysis

According to the method of Gregory et al. which has proven to be effective^30^, the normal and injured finite element models (FEM) of the porcine flexor digitorum profundus tendons were established through ABAQUS 2014. A finite element model of the tendon was established based on the experimental tensile sample. The model is an elliptical column, with semi major axis length = 3.6 mm, semi minor axis length = 2.8 mm, and height = 60 mm. One end of the model was completely fixed and the other end was displaced 15 mm outward along the tendon. The finite element model was meshed with the eight-node solid element C3D8R with a reduced integral; the element size was 0.75 mm, and the hourglass control was enhanced. The unit shape was a hexahedron, and the neutral-axis drawing method was adopted. The total number of units was 7,156. According to the fitted material parameters, the average values of six samples were selected: matrix coefficients (C1 and C2) were 24.1366 MPa and − 21.7600 Mpa, the toe region coefficient (C3) was1.95 Mpa, the rate of collagen fibre loading (C4) was 41.5, the modulus of the straightened collagen(C5) was 287.2 MPa, and the stretch value where the collagen fibers become uncrimped(λ*) was 1.067. The material density was 1.32 g/cm^3^.Yang’s modulus was 350 MPa and Poisson’s ratio was 0.3.

FEMs were used to simulate the tension of the intact tendon so that the displacement and tensile force could be compared with the intact tendon (Group C) to verify the rationality of the FEM. After verifying the rationale, FEMs were created for Groups A and B. A tendon hemisection model was used to simulate the tensile experiment. The simulated tendon stress was compared with the experimental results.

### Statistical evaluation

Statistical analysis was performed using SPSS Version 22. For Groups A and B, the failure load and length of deformation of tendons with different distances between the hemisections were compared using ANOVA. The failure load and elongation of tendons with the same distance and the farthest distance between hemisections were compared between Groups A and B using an independent sample t-test. In addition, tendons in group C were compared with all tendons in Groups A and B using a t-test to explore the effect of double or triple hemisection method on tendon biomechanics. Finally, the software (SPSS Version 22) was used to conduct motion analysis to examine the patterns of tendon lengthening during the stretching process.

## Results

### Tendon lengthening and force of different groups during the splitting

**Figure 4:**
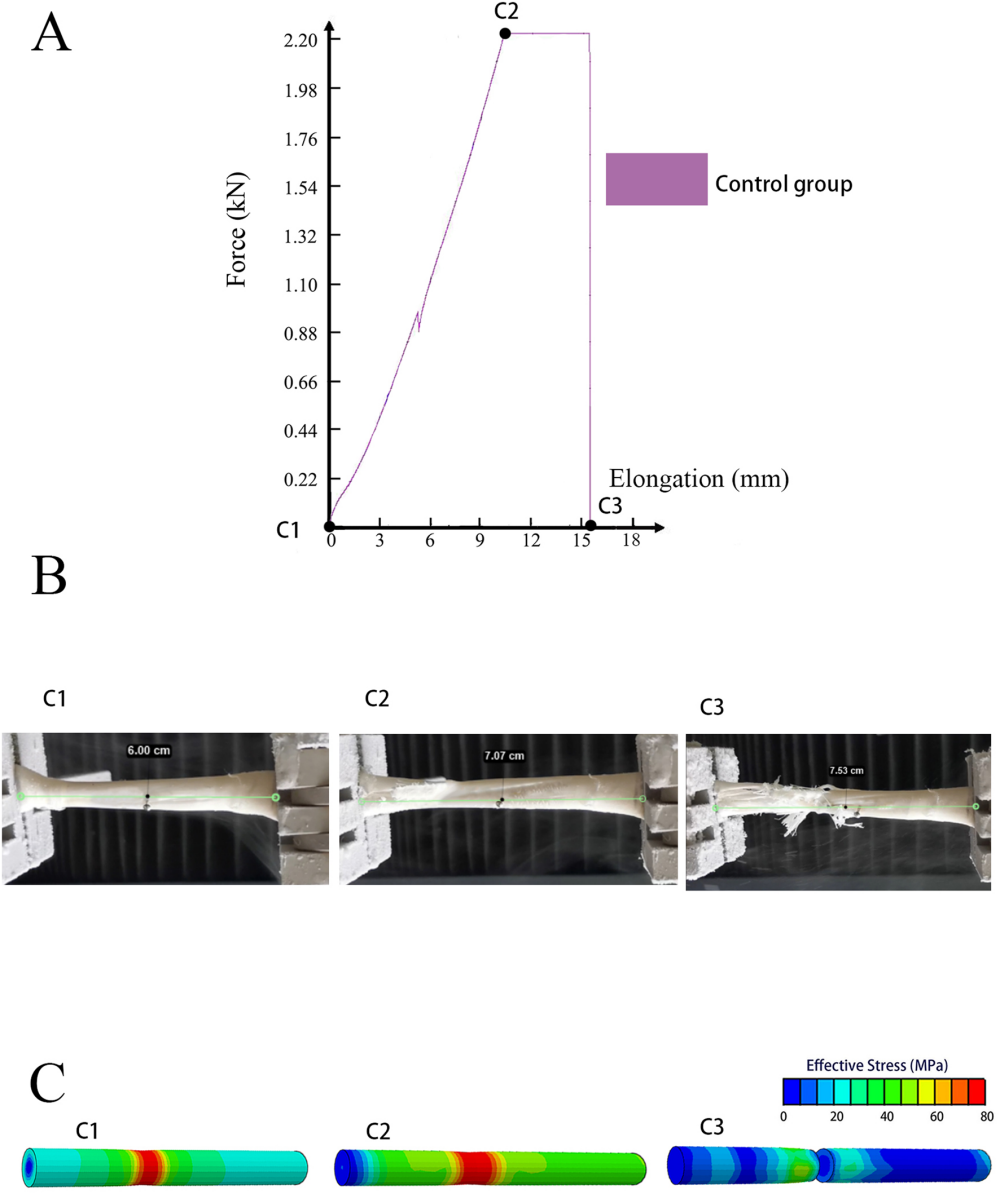
Motion analysis of the intact tendon (Group C). (**A**) The F-L curve. The black spots on the curve represent before the test (C1), when the failure load was reached (C2), and when the tendon was divided into two parts (C3). The tendon stress was gradually increased until the failure load was reached (C2). Subsequently, tendon stress was maintained at the failure load. After the tendon was elongated by approximately 5 mm, the stress rapidly decreased to zero (C3). (**B**) Video screenshots before the test (C1), when the failure load was reached (C2), and when the tendon was divided into two parts (C3). The length of the tendon test area was 6 cm before beginning the tensile test (C1). Subsequently, the tendon was lengthened at a uniform speed. When the failure load was reached, a small rupture appeared on the tendon surface (C2), followed by tendon fiber disintegration. The ruptured surface was uneven and the tendon was broken into two parts (C3). (**C**) FEM of the tendon before the test (C1), when the failure load was reached (C2), and when the tendon was nearly divided into two parts (C3). The figure shows the stress distribution in tendons. Red indicates the greatest stress, and blue indicates the lowest stress. When the tendon was stretched, the internal stress of the tendon increased, and the stress was concentrated in the test area (C2). The point with the highest stress level was the position of tendon rupture.

To explain the results more clearly, we selected one tendon each in Groups A, B, and C, and the distance between tendon incisions in Groups A and B was 1 cm. By combining the F-L curve, motion analysis, and FEM, the lengthening and stress distribution patterns of the three tendons were comprehensively analyzed. In the F-L curve of the intact tendon (one tendon in Group C as an example), the force on the tendon gradually increased during stretching, reaching a maximum force that was maintained until the tendon was ruptured (Fig. 4A). The elongation of the tendon throughout the process was caused by its deformation (Fig. 4B), and the stress was concentrated at the ruptured position (Fig. 4C). After the tendon ruptured, the force on the tendon decreased rapidly and the stress distribution disappeared (Fig. 4).

**Figure 5:**
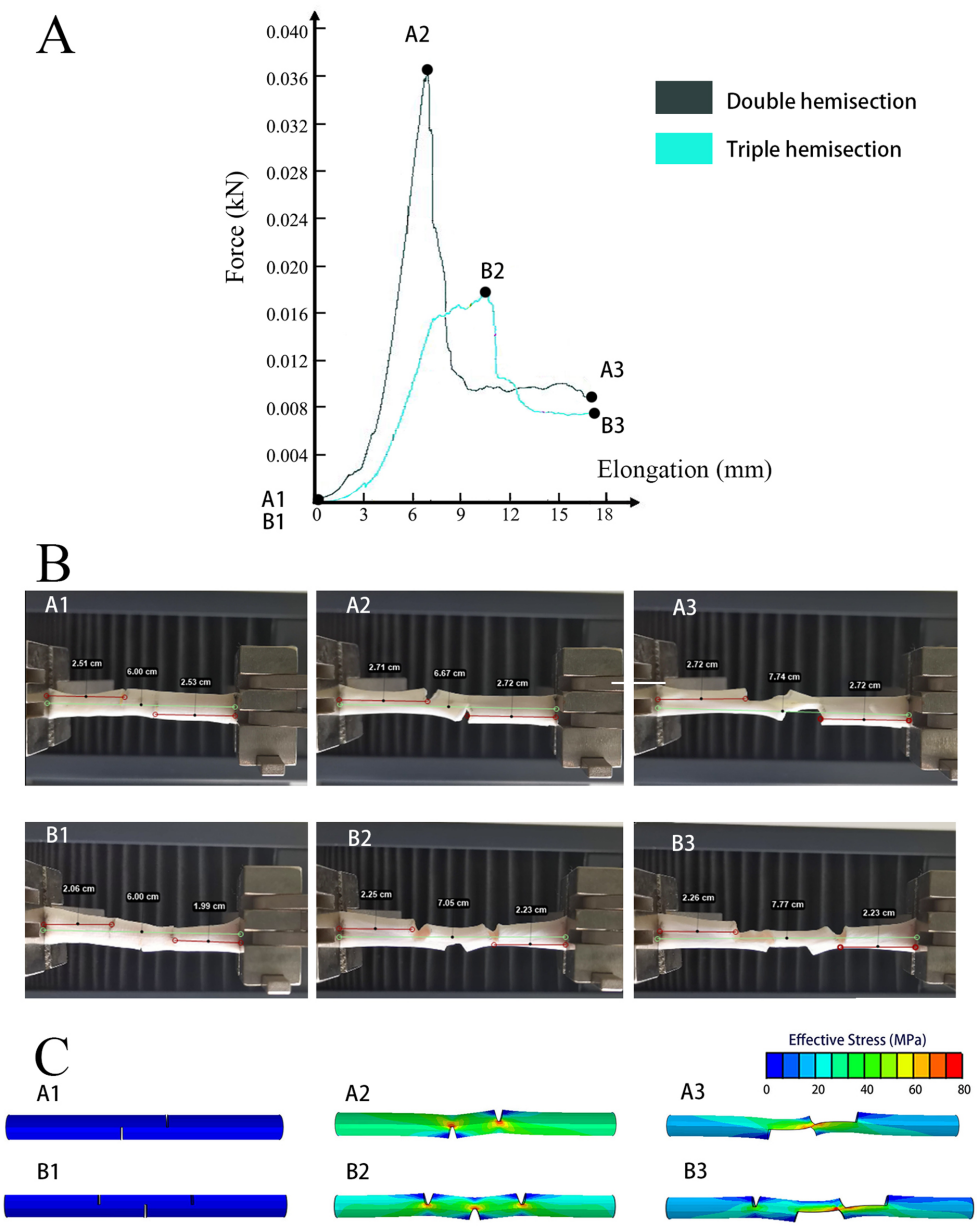
Motion analysis of tendons with double and triple hemisection treatment (Group A and Group B). The distance between sections is 1 cm. (**A**) The F-L curves. The black curve represents the double-hemisection group and the blue curve represents the triple-hemisection group. The black spots on the curve represent (A1, B1) before the test, (A2, B2) when the failure load was reached, and (A3, B3) when the tendon was divided into two parts. As the tendon stretched, the force on the tendon rapidly increased until it reached its peak (A2, B2), which was the failure load. The failure load led to the initiation of a relative split between tendon hemisections. Thereafter, the force on the tendon decreased rapidly to a force that could maintain the tendon split until the tendon almost split into two parts (A3 and B3). (**B**) Video screenshots of the double- and triple-hemisection groups before the test (A1, B1), when the tendon reached the failure load (A2, B2), and when the tendon nearly split into two parts (A3, B3). The length of the tendon test area was 6 cm before beginning the tensile test. The distance from the clamping line to the plane of the nearest hemisection was measured before and after the tendon split. There was no significant difference in the distance between the clamp line and nearest hemisection between the groups (A2 and A3; B2 and B3). (**C**) The FEM of tendons of double- and triple-hemisection groups before the test (A1, B1), when the tendon reached the failure load (A2, B2), and when the tendon nearly split into two parts (A3, B3). The figure shows the stress distribution in tendons. Red indicates the greatest stress, and blue indicates the lowest stress. When the tendon was stretched, the stress inside the tendon increased and was greatest at the hemisection site (A2, B2). With an increase in stress at the hemisection, the tendon appeared to split between hemisections, and the stress at the splitting hemisection continued to support the split further (A3, B3). The stress on the hemisections without splits decreased after the initiation of splitting in the other two hemisections, but the deformation at this non-split hemisection was maintained (B3).

The lengthening process of the tendon in the double-hemisection group (one tendon in Group A as an example) can be divided into two stages. In the first stage, the force on the tendon gradually increased to reach the failure load (Fig. 5A). During this stage, the stress at the hemisection was the highest (Fig. 5C), and the tendon hemisections were obviously deformed. With increasing stress, and after the tendon hemisections reached maximum deformation, there was a relative split between the tendon hemisections (Fig. 5B). The second stage began at this time. The force on the tendon rapidly decreased from the failure load to the maintenance force to maintain the split state of the tendon. This continued until the tendon separated into two parts (Fig. 5A). During this process, high levels of stress remained at the hemisection (Fig. 5C) to maintain the split of the tendon. It can be seen in the video that no deformation occurred between the clamping line and the hemisection, indicating that the hemisection had reached the maximum deformation before the relative split began (Fig. 5B).

The lengthening pattern of the tendons in the triple hemisection group (one tendon in Group B as an example) was similar to that in the double hemisection group, where the lengths of both the tendon deformation and split (the distance between hemisections) constituted the elongation of the tendon (Fig. 5A,B). Notably, only two hemisections in the triple hemisection group had relative splits (Fig. 5B). After the split occurred, the force on the tendon decreased and the stress was concentrated at the two hemisections where the split occurred. The stress of the third hemisection decreased (Fig. 5C), indicating that there was no chance of a split occurring in the third hemisection, which could only maintain the previous deformation. Theoretically, the elongation of the triple hemisection group should be higher than that of the double hemisection group due to the addition of extra hemisection deformation in the tendon. However, according to the results, the difference caused by the deformation of this extra hemisection was not significant (Table 1A).

**Table 1:** The elongations and failure loads with different distances between hemisections and between the most extreme hemisections in the double- and triple-hemisection groups.

(A) Elongation (cm)			
Distance between hemisections (cm)	Double hemisection	Triple hemisection	t-test
	A1	B	
0.5	1.10 (SD0.11)	1.16 (SD0.06)	0.319
1	1.69 (SD0.09)	1.73 (SD0.03)	0.353
2	2.64 (SD0.13)	2.79 (SD0.14)	0.082

Distance between most extreme hemisections (cm)	Double hemisection	Triple hemisection	t-test
	A2	B	
1	1.69 (SD0.09)	1.16 (SD0.06)	0.000*
2	2.64 (SD0.13)	1.73 (SD0.03)	0.000*
4	4.63 (SD0.06)	2.79 (SD0.14)	0.000*

(B) Failure load (N)			
Distance between hemisections (cm)	Double hemisection	Triple hemisection	t-test
	A1	B	
0.5	19.24 (SD7.15)	9.74 (SD3.53)	0.021*
1	27.47 (SD6.14)	18.62 (SD5.65)	0.027*
2	44.56 (SD5.60)	58.14 (SD17.10)	0.114

Distance between most extreme hemisections (cm)	Double hemisection	Triple hemisection	t-test
	A2	B	
1	27.47 (SD6.14)	9.74 (SD3.53)	0.000*
2	44.56 (SD5.60)	18.62 (SD5.65)	0.000*
4	388.26 (SD108.96)	58.14 (SD17.10)	0.001*

(A) *P < 0.05. Elongation of tendons in the double-hemisection group was significantly higher than that in the triple-hemisection group when the distance between the most extreme hemisections was 1 cm, 2 cm or 4 cm.

(B) *P < 0.05.: Failure load of tendons in the double-hemisection group was significantly higher than that in the triple-hemisection group when the distance between hemisections was 0.5 cm or 1 cm, or the distance between the most extreme hemisections was 1 cm, 2 cm or 4 cm.

### Elongation and failure load of tendons in double and triple hemisection groups compared with intact tendons (Groups A and B versus Group C)

**Figure 6:**
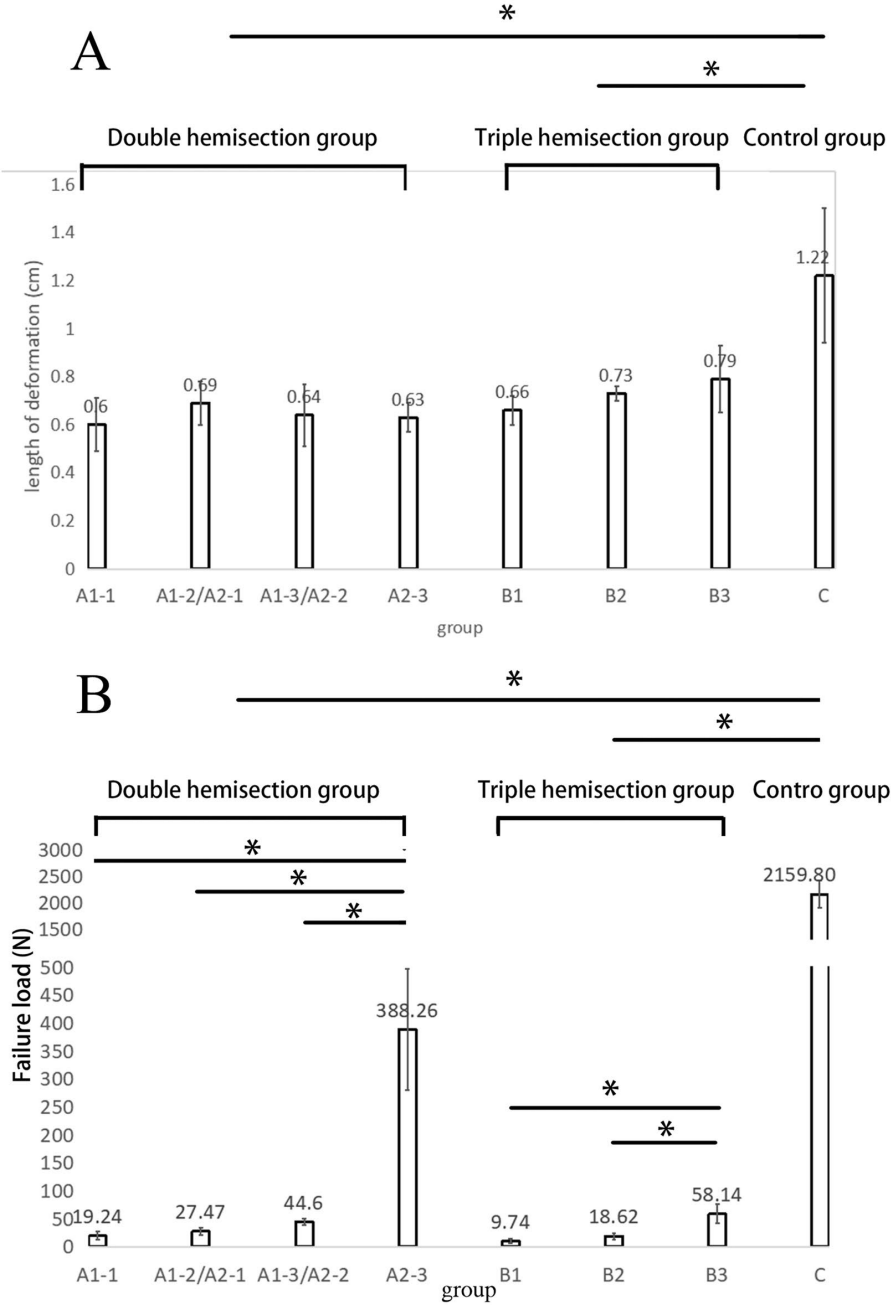
(**A**) Length of the deformation of tendons and (**B**) failure load with different treatments. (**A**) The length of the deformations of intact tendons (Group C) was significantly higher than tendons in the double- and triple-hemisection groups (Groups A and B). (**B**) *P < 0.05. Failure load of tendons in A2-3 were significantly higher than other sub-groups of the double-hemisection group. The failure load of tendons in B3 was significantly higher than that of tendons in B1 and B2 in the triple-hemisection group. The failure load of tendons in Group C was significantly higher than that of tendons in Groups A and B.

The failure load of tendons with double and triple hemisections was significantly lower than that of intact tendons (P < 0.05, Fig. 6B). When the distance between the hemisections was less than 2 cm, there was no significant difference in elongation between the double or triple hemisection tendons and the intact tendons (Table 2). When the distance between the hemisections reached 2 m and 4 cm, the elongation of double or triple hemisection tendons was significantly higher than that of intact tendons (P < 0.05, Table 2).

**Table 2:** Comparison of the elongation of tendons in the double- and triple-hemisection groups (Groups A and B) compared with intact tendons (Group C).

Group	Distance between hemisections (cm)	Elongation (cm)	P value against C
Double hemisection (A)	0.5	1.10 (SD0.11)	0.386
	1	1.69 (SD0.09)	0.008*
	2	2.64 (SD0.13)	0.000*
	4	4.63(SD0.06)	0.000*
Triple hemisection (B)	0.5	1.16 (SD0.06)	0.631
	1	1.72 (SD0.03)	0.006*
	2	2.79 (SD0.13)	0.000*

The elongation of intact tendon (in group C) was 1.22 (SD0.28) cm.

*P < 0.05. The elongation of hemisected tendons where the distance between hemisections was over 1 cm (Groups A and B) was significantly higher than that in intact tendons (Group C).

### The effect of distance between hemisections on elongation and failure load (Group A or Group B)

The length of the deformation of each group was calculated according to the tendon lengthening pattern mentioned above, where tendon elongation consists of the length of the deformation and the splitting distance between hemisections. For Group C, this was simply the elongation of the tendons. For Groups A and B, this was the elongation minus the distance between the hemisections. The results showed that the length of the deformation of intact tendons was significantly higher than that of tendons in the double- and triple-hemisection groups (Fig. 6A). Accordingly, the potential for tendon elongation was determined by the distance between hemisections.

With an increase in the distance between hemisections, the failure load of the tendon also increased. In the double-hemisection group, the failure load of the tendon was not significantly different when the distance between the hemisections was 0.5, 1, or 2 cm (Fig. 6B). When the distance between the hemisections reached 4 cm, the failure load of the tendon increased significantly (P < 0.05) (Fig. 6B). In the triple hemisection group, the failure load of tendons with a distance of 2 cm between hemisections was significantly higher than that of tendons with other distances (P < 0.05, Fig. 6B).

### The difference in elongation and failure load between double- and triple-hemisection groups (Group A versus Group B)

When the distance between the hemisections was the same, the difference between the elongation tendons in the double- and triple-hemisection groups was not significant (Table 1A). When the distance between the hemisections was 0.5 cm or 1 cm, the failure load of the tendons in the triple hemisection group was significantly lower than that of the tendons in the double hemisection group (P < 0.05, Table 1B).

Additionally, when the distance between the most extreme hemisections was the same, the elongation and failure load of the tendons in the triple hemisection group were significantly lower than those in the double hemisection group (P < 0.05, Table 1).

## Discussion

Slide tendon lengthening is an effective method for the treatment of limb contractures caused by long-term spastic paralysis. The disadvantages of Z-lengthening, such as larger incisions and greater damage to the tendons, have been previously reported. However, such disadvantages appear to occur more frequently when the deformity is severe^21,22^. Hashimoto et al.^19^ reported that slide lengthening can provide a higher ultimate tensile strength than Z-lengthening. However, the optimal number of hemisection planes and the optimal distance between hemisection planes for slide lengthening have not been explored. In the current study, the length of the flexor digitorum profundus harvested from the hind legs of the pigs was limited (less then 10 cm). Accordingly, double and triple hemisections were used, which are both relatively common methods reported in previous studies^8–11,17,19,20^.

The failure load is the force to which the tendon is subjected when it splits or ruptures. Biomechanical analysis showed that the failure load of tendons was significantly reduced following double or triple hemisection treatments compared with untreated tendons. In addition, greater distances between hemisections lead to higher failure loads. Greater elongation of the tendon was observed in the double- and triple-hemisection groups. The current study found that the failure load increased significantly when the distance between hemisections was 4 cm, increasing sharply to 388.26 ± 108.96 N, which was eight times higher than that of the groups with distances less than 2 cm. However, when the distance between the hemisections was 2 cm, the failure load of the tendons in the double- and triple-hemisection groups was not significantly different. Therefore, for patients with tendon elongation, double hemisections could meet the demand through a reasonable design of the hemisection location. This leads to fewer incisions. Therefore, double hemisections are more likely to be selected than triple hemisections.

The results of the current study showed that the elongation of tendons that underwent double or triple hemisections correlated with tendon deformation and the distance between the hemisections. However, there was no significant difference in the length of deformation and elongation of the tendon between the double- and triple-hemisection groups with the same distance between the hemisections. This lack of difference could be due to the slight deformation and lack of splitting in one of the hemisections in the triple hemisection tendons, which was observed in both the motion and finite element analyses. In addition, in the finite element analysis, the level of stress at the non-split hemisection in triple-hemisection tendons decreased after splitting of the other two hemisections was initiated. The decreased stress, which could not maintain the maximum deformation length or initiate split lengthening, could be the reason for the lack of deformation and splitting. Furthermore, this could also explain why the double hemisections (A2 group) showed greater elongation ability than the triple hemisections (B group), even when the distance between the most extreme hemisections was the same. The mechanism of non-synchronized splits in hemisections and a method to prevent them from occurring should be explored in future studies. In addition, the elongation of intact tendons caused by elastic deformation might limit the significance of clinical tendon lengthening, although the elongation of the intact tendon was significantly higher than that of tendons that underwent double or triple hemisections when the distance between the hemisections was less than 2 cm (Table 2).

**Figure 7:**
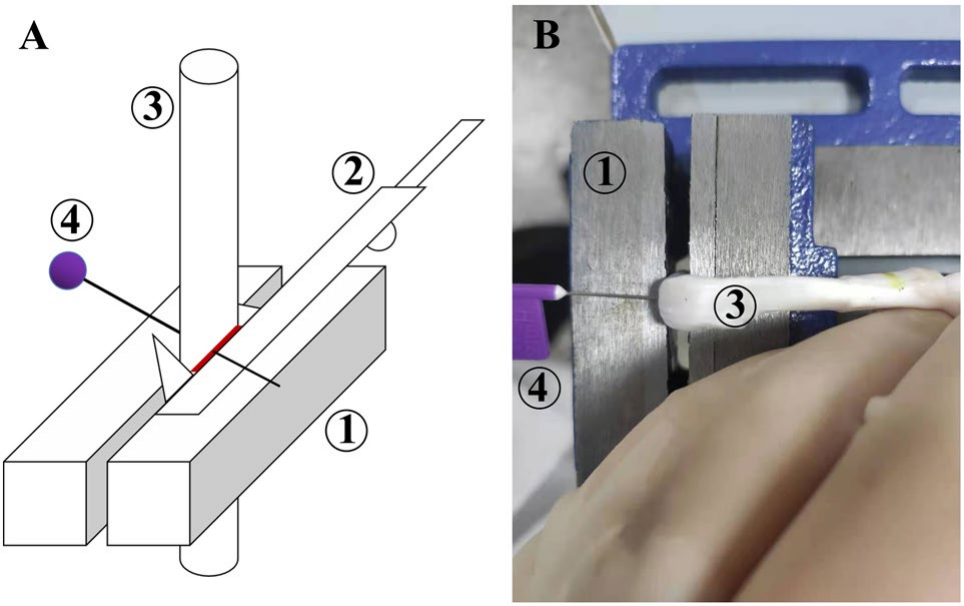
Establishment of tendon hemisections. (**A**) Pattern diagram. (**B**) Realistic operation. (1) Flat clamp. (2) Vernier caliper. (3) Tendon. (4) Marker. The flat clamp compressed the tendon along the hemisection plane, and the cross-sectional area of the tendon was approximately rectangular. The width of the area on the contact side was measured with a clamp using a Vernier caliper. A marker was inserted at the midpoint of the width. Subsequently, a surgical blade was used to cut the tendon perpendicular to the clamped surface. When the midpoint was reached, the marker fell.

Attention should be paid to the size of tendon hemisections. If the section is too small, less than half of the cross-sectional area of the tendon, intact tendon fibers between the sections maintain the integrity of the tendon, even when the two sections are parallel. This can result in inaccurate measurement. If the half section is not perpendicular to the tendon, a larger section is required to achieve the half-cut effect. Furthermore, the angle of incision affects the tendon^22^. If the half-sections do not have an opposite relationship, it may result in rotation of the tendon and avulsion, caused by the stretching angle between the incisions. This in turn can affect the biomechanical properties of tendons. Therefore, to achieve a unified test environment, a flat clamp was used as an auxiliary. The flat clamp was compressed slightly at the hemisection plane, with the upper edge of the clamp aligned to the plane. At that time, the cross-sectional area of the tendon in the clamp was almost rectangular in shape. This can then be measured on the long side and the midpoint marked. The tendon was then carefully cut along the upper edge of the flat clamp perpendicular to the long axis of the tendon until the midpoint was reached (Fig. 7). Slight compression of the tendon had no effect on its biomechanical properties^31^.

The current study has several limitations. First, although the software used was reliable video analysis software^32^ that is commonly used to conduct motion analysis and clinical analysis and to evaluate the reliability of other new technologies^32^, it requires the operator to mark the measurement range in the video interface. Therefore, errors were possible. Second, the cross-sectional areas of tendons vary when the tendons have different lengths^33^. This can lead to different hemisection locations, which may have introduced bias into the results. Third, although the anatomy of the porcine flexor tendon system is similar to that of a human digit^24^, differences between the two species may impact the generalizability of these findings. Finally, there is clinical guidance for in vitro experiments compared with in vivo experiments. The tendon in vivo has its own healing and adhesion properties with the surrounding tissues after hemisection. The impact of the operation on these properties is difficult to predict. Furthermore, the in vivo environment is difficult to simulate in an in vitro setting. Therefore, in vivo experiments are of clinical significance. Future research could focus on in vivo experiments to improve the clinical significance of these results.

## Conclusions

From the results of this study, it was found that the lengthening ability of the double and triple hemisections, with the same distance between hemisections, was similar. However, the lengthening ability of the double hemisections was better when the distances between extreme hemisections remained the same. Tendon lengthening in double hemisections may require a higher driving force to initiate lengthening, which should be considered in the design of clinical surgery, especially in patients that require longer tendon lengthening.

## Data Availability

The datasets generated during and/or analysed during the current study are available from the corresponding author on reasonable request.
